# 

**Published:** 1889-12

**Authors:** 


### CONTENTS.

### COMMUNICATIONS.

Address.............................................................................. 577
A Study of the Chemical Composition of the Dental Pulp.............. 581
Womens' Work in the Profession........................................... 586
One way of Increasing a Dentist's Usefulness.......................... 589
Failures ............................................................................... 592
Excelsior ............................................................................. 595
Report of Seventh District Ohio Dental Society........................ 597
The Disputes Between the Editors of the Dental Journals............. 599
Abortive Abscesses............................................................. 600
Proceedings.......................................................................... 601

### SELECTIONS.

Death Under Anæsthetics...................................................... 609
Opinion of the Medical Press upon the Death Under Nitrous Oxide 610
Anæsthetics and Accidents.................................................... 613
Chloroform as an Anæsthetic................................................. 615
Anæsthesia .......................................................................... 616
Preservation of Colored Anatomical Specimens......................... 618
Pea Soup as a Substitute for Beef Tea...................................... 619
Preparing and Mounting Sections of Brain................................ 619
Overcrowding of the Professions in Germany............................ 620

### NOTICE.

The International Medical Congress of 1889.............................. 621

### EDITORIAL.

Close of the Volume............................................................ 622
Books.................................................................................. 623

